# Neurodevelopmental Impact of Maternal Postnatal Depression: A Systematic Review of EEG Biomarkers in Infants

**DOI:** 10.3390/children12040396

**Published:** 2025-03-21

**Authors:** Roxana Şipoş, Iulia Calugar, Elena Predescu

**Affiliations:** 1 Department of Neuroscience, Psychiatry and Pediatric Psychiatry, “Iuliu Hatieganu” University of Medicine and Pharmacy, Calea Manastur Street No. 54C, 400658 Cluj-Napoca, Romania; 2 Department of Clinical Psychology and Psychotherapy, Faculty of Psychology and Educational Sciences, Babeş-Bolyai University, 37 Republicii Street, 400015 Cluj-Napoca, Romania

**Keywords:** postpartum depression, infant neurodevelopment, EEG biomarkers, electroencephalography, maternal mental health

## Abstract

Background/Objectives: Postpartum depression (PPD) significantly impacts maternal well-being and child neurodevelopment. While the etiology of PPD is well understood, the precise neurodevelopmental consequences, particularly differentiating prenatal and postnatal effects, remain unclear. This systematic review aims to synthesize the existing literature on the neurophysiological effects of maternal PPD on infant neurodevelopment, focusing on electroencephalography (EEG) biomarkers to identify consistent patterns and potential mediating factors. Methods: A comprehensive literature search across PubMed/MEDLINE, Web of Science, and Scopus identified studies investigating infants (0–12 months) exposed to maternal depressive symptoms (assessed via validated psychometric instruments) with quantitative EEG data. Study quality was assessed using the Newcastle–Ottawa Scale. Results: Twelve studies met the inclusion criteria. Eleven investigated EEG asymmetry, predominantly frontal alpha asymmetry (FAA). The findings consistently showed greater right FAA in the infants of mothers with PPD, suggesting increased negative affectivity and avoidance behaviors. This association was stronger with prolonged or combined prenatal/postnatal exposure. However, EEG power and connectivity findings were less consistent, with some studies reporting altered occipital power at 1 month and frontal power at 3 months in the infants of depressed mothers. No significant associations were found between maternal depression and functional connectivity. Conclusions: This review demonstrates a robust association between maternal PPD and altered infant EEG patterns, particularly increased right FAA. However, methodological heterogeneity necessitates future research with standardized protocols and longitudinal designs to establish causality and investigate long-term effects. Further research should also explore the underlying neural mechanisms and evaluate the efficacy of targeted interventions. These findings underscore the need for early identification and intervention to mitigate the negative impact of PPD on infant neurodevelopment.

## 1. Introduction

Rooted in dynamic interactions between hormonal dysregulation [1,2], genetic predispositions [3], and environmental stressors [4], postpartum depression (PPD) represents a distinct neuropsychiatric phenomenon with implications for both maternal functioning as well as child development [5]. In recent years, PPD has emerged as a significant public health problem, affecting approximately 17% of individuals worldwide [6]. Aside from its prevalence and multifaceted etiology, PPD stands out as one of the most debilitating postpartum complications, characterized by symptoms such as persistent sadness, fatigue, impaired concentration, sleep disturbances, and feelings of inadequacy or hopelessness [7]. If left untreated, postnatal depression can hinder the ability to care for the infant and may contribute to long-term negative outcomes in child development beyond early infancy.

Early childhood, particularly infancy, represents a critical period for neural development due to rapid brain maturation, heightened synaptic plasticity, and the foundational establishment of cognitive and emotional regulation [8]. Infants of mothers experiencing PPD are particularly vulnerable to emotional, behavioral, and psychological problems, as well as delays in cognitive and language development that can extend into adolescence [9,10]. Regarding the mechanisms through which maternal depression exposure, both antenatal and postnatal, may influence offspring brain development, several hypotheses have been proposed, although the precise pathways remain to be fully elucidated. From a biological perspective, several mechanisms may underlie the link between maternal depression and infant neurodevelopment. During pregnancy, hypothalamic–pituitary–adrenal (HPA) axis dysregulation characterized by prolonged elevations of cortisol, elevated norepinephrine, and decreased serotonin can directly affect fetus development via the maternal–placental–fetal neuroendocrine axis [11,12]. Furthermore, infants exposed to postnatal depression have been shown to exhibit higher cortisol levels compared to infants whose mothers are not depressed [11]. Data have indicated that elevated levels of glucocorticoids persist into childhood and even adolescence, underlying the long-term impact of early adverse experiences [13]. Moreover, epigenetic modifications, altered by exposure to significant stress early in life, may lead to offspring HPA axis ‘reprogramming’ and dysregulation due to the DNA methylation of genes. These changes have been observed not only in infants exposed to antenatal depression but also in those exposed exclusively to postnatal depression [14]. Furthermore, changes in neurotrophic factor signaling may impact infants of depressed mothers. Deficiencies in maternal brain-derived neurotrophic factor (BDNF) during pregnancy have been linked with gestational abnormalities, such as intrauterine growth restriction, preterm birth, and low birth weight, all of which could pose significant long-term impacts [15]. Another theory suggests that the quality of the mother–infant interaction plays a crucial mediating role [16]. Evidence indicates that mother–child interactions are often negatively impacted during early maternal care. These findings suggest that depressed mothers initiate less physical contact with their infants, which is often characterized by a less affectionate style of touch [17,18]. Furthermore, it has been demonstrated that they exhibit disengaged behaviors [19], displaying a decreased use of infant-directed speech, smiling, and vocal communication engagement, which can lead to language development problems [20]. A meta-analysis of 14 studies investigating child cognitive development demonstrated a significant association between maternal depressive symptoms and lower cognitive scores in infants and young children under 56 months of age [21].

Another hypothesis posits disruptions in neural pathways and connectivity, which may underline the observed effects of maternal postnatal depression on infant development. Research has shown altered brain circuitry across multiple functional neuroimaging studies [22,23]. A longitudinal functional neuroimaging fMRI study indicated that boys who have been exposed to maternal depression during early childhood exhibit modified reward processing. Specifically, these boys may demonstrate altered activation patterns in brain regions associated with reward, such as the medial prefrontal cortex and striatum, when anticipating and experiencing reward and loss [24]. Nonetheless, a more recent study, however, found limited evidence of differences in functional connectivity in reward tasks between the offspring of mothers with depression and those without [25].

In addition, neurophysiological studies using electroencephalography (EEG) have provided further insights into the neural effects of maternal depression on infant brain development. Early research in this area has primarily focused on asymmetries in alpha power, particularly between the right and left hemispheres (FAA). FAA is indicated to be a reliable comparative metric, showing high internal consistency reliability and strong test–retest reliability over time intervals of several months [26]. It has been associated with approach/withdraw emotional tendencies and motivational processes [27,28]. Infants exposed to prenatal maternal depression exhibit greater relative right frontal alpha asymmetry, which is thought to reflect increased susceptibility for negative affectivity and avoidance behaviors [29,30]. An earlier review [31] highlighted findings of greater relative right frontal EEG asymmetry during a resting state in the infant offspring of depressed mothers, although the distinction between the effects of prenatal and postpartum depression on the observed asymmetry was not explicitly addressed in the review. Furthermore, more findings on frontal alpha asymmetry in infants have suggested that greater right frontal alpha asymmetry is linked to less positive mother–infant interactions during adversity [32], as well as a higher risk of externalizing and internalizing problems in later years into childhood [33,34]. Additionally, findings in neurophysiological studies also reveal developmental changes in neural activity, including shifts in alpha power from posterior to anterior brain regions, which parallel advances in both cognitive and emotional regulation capacities [35]. Developmental transitions from low-frequency to higher-frequency power in infant EEG signals are indicative of neural maturation, with indicators such as the alpha-to-delta power ratio [36,37] and delta–beta coupling [38] serving as markers of more advanced neurodevelopment. It has been found that a low frontal alpha-to-delta ratio correlates with higher levels of inhibition and social avoidance by age 10 years [36], as well as higher levels of social anxiety in adolescence [39]. Similarly, greater delta–beta coupling—a marker of correlation of neural activity between slow-wave oscillations (delta) and fast wave oscillations (beta)—has been associated with higher levels of fear, shyness [40], and a greater risk of anxiety in children [41]. Thus, the maturational changes indicated are associated with both emotional and cognitive development [36], suggesting that maternal depression may compromise the typical maturation of neural systems that underpin adaptive emotional and cognitive processes in infants.

Despite the significant body of research on maternal depression and its impact on neurodevelopment, significant gaps remain. Notably, much of the current literature has not adequately distinguished between the effects of prenatal depression and postpartum depression. This lack of clarity complicates our understanding of whether the observed neurophysiological changes in infants can be attributed solely to PPD or are influenced by a combination of prenatal and postnatal factors. Additionally, many studies fail to account for potential confounders, such as maternal anxiety, breastfeeding practices, or socioeconomic factors, which could also shape infant neurodevelopment.

As a result, it remains unclear whether the observed changes in neurophysiological activity, as measured by EEG, can be exclusively attributed solely to PPD or whether they reflect the combined influence of prenatal and postpartum factors.

To address this gap in the literature, we are conducting this systematic review to synthesize the existing literature on the neurophysiological effects of maternal postnatal depression on infant neurodevelopment, specifically focusing on EEG biomarkers to identify consistent patterns and potential mediating factors.

## 2. Materials and Methods

### 2.1. Search Strategy

The current review was conducted in line with the PRISMA guidelines for systematic reviews and meta-analyses [42]. A comprehensive literature search was carried out across three major electronic databases: PubMed/MEDLINE, Web of Science, and Scopus. Search strings were tailored to each database and utilized controlled terms related to infant neurophysiological data and maternal depression exposure. Full search strategies for each database are available in Supplementary File S1.

### 2.2. Inclusion Criteria

Inclusion criteria were established by employing an approach based on the PICOS principles [43]. Studies would be included if they had investigated both infants aged 0–12 months who had been exposed to maternal depressive symptoms during their first year of life as well as their mothers. This age range was chosen as it represents a critical window for early neurodevelopment and caregiver–infant bonding. While a formal clinical diagnosis based on the DSM or ICD criteria is not required, maternal depression must be assessed using established psychometric instruments for assessing depression. Included studies had to utilize neurophysiological EEG assessments of infants and report quantitative EEG data. If studies had investigated both prenatal and postnatal maternal depression, they would be included only if they had provided separate data for these two exposure types. Eligible studies had to be peer-reviewed articles, published in English, with study designs including both observational (cohort, case–control, cross-sectional, etc.) and interventional types. 

### 2.3. Study Selection

**Figure 1:**
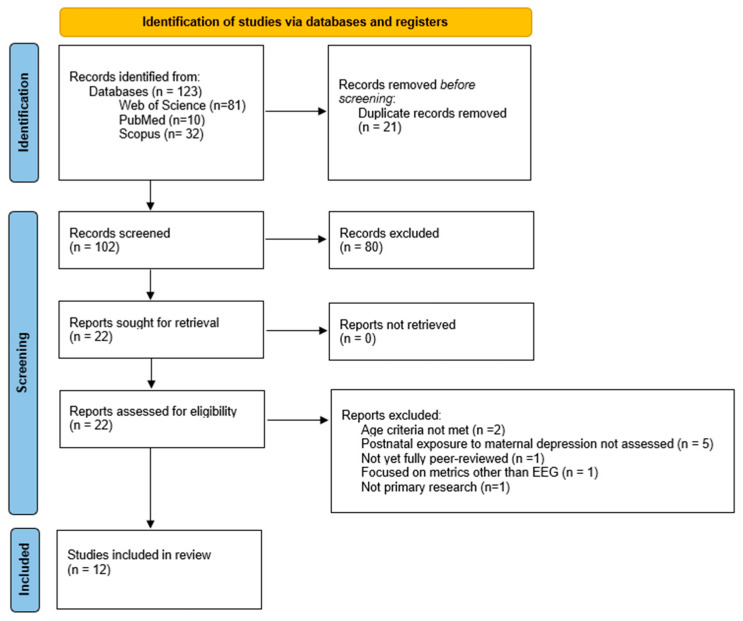
PRISMA flow diagram.

The literature search was conducted by a single reviewer in January 2025, yielding 123 records. Following duplicate removal, 102 records were screened by title and abstract against the inclusion and exclusion criteria using the Zotero reference management software (v4.0), which led to 80 excluded records. Thus, 22 records were identified for full-text review and were evaluated for eligibility by the first and second reviewers. Any disagreements between the reviewers were resolved through discussion and, if necessary, the involvement of the third reviewer. Out of the 22 records, 10 were excluded for the subsequent reasons: 2 studies did not meet the participants’ age requirement [44,45], 5 studies had not assessed postnatal exposure to maternal depressive symptoms [46,47,48,49,50], 1 study had focused on physiological metrics other than EEG data [51], 1 study had not yet been fully peer-reviewed [52], and 1 was not an original empirical study [53]. The selection process is also illustrated in Figure 1.

### 2.4. Quality Assessment and Interrater Reliability

Two reviewers independently assessed the quality of the studies using the Newcastle–Ottawa Scale (NOS) for non-randomized case–controls and cohort studies. This quality assessment employed rating the studies on three domains—group selection, group comparability, and the determination of exposure/outcome of interest—assigning scores on a 0–9 scale using a star-based system [54]. Any interrater discrepancies were resolved through consensus discussion (see Supplementary File S2). 

### 2.5. Data Extraction and Analysis

Data were extracted from the included studies in relation to author and year of publication, design type, population (sample size, mother and infant mean age, infant gender, mother marital status, parity), maternal depression exposure (type of exposure, measures) EEG recording methodology (acquisition procedure, reference, and epoch duration), and reported EEG findings. These data were organized into a singular database using IBM SPSS Statistics software V.17 and descriptive statistics were calculated for key sample characteristics. For case–control studies reporting demographic data stratified by subgroups, weighted means were calculated for both case and control groups to derive overall estimates. Specifically, for one of the included studies [55], maternal age was reported as means within subgroups defined by both depression status (depressed/non-depressed) and infant feeding method (breastfed/bottle-fed); overall estimates were derived for the depressed and non-depressed groups using weighted means and pooled standard deviations [56]. This approach ensured improved data homogeneity and accurately reported overall means.

Notably, two of the included studies reported data on a partially overlapping sample of infants [57,58]. However, both studies have been retained in our review as they provided data at different developmental ages. Lusby et al. (2014) [57] provided data at 3 months and 6 months of age while Goodman et al. (2021) [58] reported data on the same sample at 12 months of age. To uphold the integrity of our review, we have thoroughly extracted and documented the data from each study separately.

## 3. Results

### 3.1. Overview and Sample Characteristics of Included Studies

A total of 12 studies were deemed eligible for inclusion in this analysis. Out of the twelve studies, two of them employed a case–control design while ten utilized a cohort design. The majority of the studies (n = 8) were conducted in the United States of America while the remaining studies included participants from Singapore (n = 2), Canada (n = 1), and Italy (n = 1).

The studies investigated mother–infant dyads, with sample sizes ranging from 65 to 258 dyads. Weighted means and pooled standard deviations were computed for maternal age parameters, yielding a mean age of M = 31.48 years (SD = 4.97) across the 11 studies that reported sample characteristics. Based on the available data, 84.43% of the mothers were married (n = 9 records), and 42.9% were primiparous (n = 6 records). Infants included in the studies ranged from 1.7 weeks of age to 12 months at enrollment, with approximately 49.66% identified as male (n = 10 records). The study and sample characteristics for each study are enlisted in Table 1.

### 3.2. Exposure to Maternal Depression 

All included studies measured depressive symptoms in the postnatal period while 8 of them also measured maternal depressive symptoms during pregnancy. Data on maternal depression were obtained exclusively through self-report instruments. Different psychometric tools were utilized across the included studies as summarized in Table 2.

The assessment of depressive symptoms in mothers included a variety of reliable psychometric tools. Most studies had utilized well-established scales. The Center for Epidemiologic Studies Depression Scale (CES-D) consists of 20 items designed to measure depressive symptoms in the general population. The CES-D has demonstrated strong internal consistency (Cronbach’s alpha ranging from 0.85 to 0.90) and validity in various populations, making it a widely used instrument for detecting depression in both prenatal and postnatal contexts [67,68]. The Beck Depression Inventory (BDI) and BDI-II are 21-item self-report instruments that assess the presence and severity of depressive symptoms. The BDI shows excellent reliability, with a Cronbach’s alpha of approximately 0.91 for the BDI-II [69]. These measures are frequently utilized in clinical settings for their established validity in detecting depressive symptoms among postpartum mothers [70]. The Edinburgh Postnatal Depression Scale (EPDS) comprises 10 items and is specifically designed for screening postpartum depression. It has been validated for use in various populations, exhibiting good reliability, with Cronbach’s alpha values typically around 0.87 [71]. The EPDS is regarded as a reliable tool for identifying women at risk of postnatal depression and has been validated across multiple cultural contexts [72].

### 3.3. Neurophysiological EEG Metrics

The neurophysiological assessment of infants exposed to maternal depression was a focal point in the majority of the included studies. Specifically, 11 studies investigated EEG asymmetry, which reflects differential brain activity between hemispheres. Additionally, some studies reported EEG power and coherence, while only one study focused specifically on the alpha–delta ratio (see Table 3).

The neurophysiological metrics assessed varied widely across studies, with a predominant focus on EEG asymmetry, highlighting its potential role as a biomarker for understanding the impact of maternal depression on infant brain development. These metrics provide insights into the asymmetric activation of brain regions associated with emotional and cognitive processes. Table 3 presents a comprehensive overview of EEG acquisition parameters, included metrics, and key findings from each study. Overall, the findings suggest that infants exposed to maternal depression exhibit greater right frontal EEG asymmetry, potentially indicating an increased susceptibility to negative emotional states. Additionally, notable variations across different caregiving contexts, such as breastfeeding versus bottle-feeding, appear to influence EEG outcomes. For instance, Hardin et al. (2021) [60] reported varying results in EEG power and asymmetry based on feeding methods. This variability underscores the importance of considering environmental and maternal behaviors when interpreting neurophysiological outcomes.

## 4. Discussion

This systematic review has investigated the neurodevelopmental impact of maternal postnatal depression on infants, focusing on EEG biomarkers.

The characteristics of the included studies (Table 1) demonstrated a diverse representation of mother–infant dyads across different countries. Furthermore, the studies provided a comprehensive overview of maternal age, marital status, parity, infant age at enrolment, and gender distribution, which may help contextualize the findings related to maternal depression and infant neurodevelopment.

Across the included studies, maternal depressive symptoms were measured at different time points and on varying sample populations (Table 2). Notably, the scores indicated that mothers often experience symptomatic fluctuations between the prenatal and postnatal periods. For example, Diego et al. (2004) [11] reported a significant difference between the prepartum (25.75) and postpartum (23.30) groups, highlighting a persistent prevalence of depressive symptoms even after childbirth. Across several studies, the total maternal depressive symptom scores revealed that even during the postnatal phase, mothers exhibit moderate levels of depression, which can have profound implications for their interactions with their infants. This consistent measurement of depressive symptoms throughout both prenatal and postnatal periods underscores the necessity to understand the full spectrum of maternal mental health in relation to infant neurodevelopment.

The majority of our findings regarding EEG measures demonstrated an association between maternal PPD and altered neurophysiological patterns in infants, particularly concerning EEG asymmetry, power, and connectivity. These findings aligned with and extended upon previous research highlighting the significant influence of maternal mental health on early brain development. However, we acknowledge that the literature presents mixed findings with some studies attributing these associations to prenatal or dual-exposure, rather than postnatal exposure only.

### 4.1. EEG Asymmetry

The preponderance of studies in this review examined EEG asymmetry, with a primary focus on frontal alpha asymmetry (FAA) and parietal alpha asymmetry (PAA). FAA is a well-established indicator of emotional regulation and approach–withdrawal tendencies. Our findings showed a trend towards greater relative right frontal alpha asymmetry in the infants of depressed mothers, reflecting increased susceptibility for negative affectivity and avoidance behaviors. Across the included studies, we observed that higher levels of right FAA were associated with prolonged exposure to maternal depression. This could have been due to the cumulative effect of prenatal and postnatal exposure, where the neural changes initiated in utero may have been further reinforced by early-life adversities.

Thus, alterations in EEG asymmetry were particularly evident in contexts of both prenatal and postnatal exposure, characterized by greater FAA [11,57,58]. Additionally, atypical shifts in EEG asymmetry trajectories were noted with extended exposure periods [63,65]. The prenatal exposure component was highly prevalent across studies. Findings of multiple studies suggest that elevated FAA in infants is more strongly associated with prenatal influences rather than postnatal ones [11,57,58]. However, all included studies that investigated exclusively postnatal depressive symptoms consistently reported greater right FAA in infants of mothers exhibiting symptoms of PPD [55,59,60,64]. Interestingly, findings were more mixed in studies examining dual exposure, with some studies reporting no significant association between offspring FAA and postnatal depression. Significant associations were found between greater relative right FAA asymmetry and prenatal depression among women with an increase of depressive symptoms during the postnatal period [57,65]. The stability of these associations is questionable, as one study reported higher right FAA at 6 months but not at 18 months of age [65]. Furthermore, our findings align with extensive research that has established mother–infant interaction as a critical key mechanism underlying child development [73,74,75]. Specifically, it has been indicated that infants of depressed mothers who have been breastfed [55,60] or experienced positive maternal sensitivity during play [66] or whose mothers have exhibited higher interaction involvement [59] do not exhibit elevated right FAA scores. In contrast, infants who have been bottle-fed [55,60] and whose depressed mothers have displayed withdrawn behaviors [59] are associated with greater right FAA. This suggests a possible mechanism by which maternal PPD might influence early emotional development in infants. The observed differences in FAA persisted even when controlling for factors such as infant age and feeding method in some studies, strengthening the robustness of the association.

Moreover, frontal alpha asymmetry (FAA) has been identified as a stable marker over time. Our findings, which indicate greater right FAA in children exposed to maternal depression, suggest that these children may experience long-term effects. Right FAA has been associated with withdrawal-related personality traits, such as shyness, the avoidance of social situations, and fearful behaviors, thereby increasing the susceptibility of these children to psychopathological outcomes [76].

Another EEG asymmetry marker of interest, which was delineated by our findings, is parietal alpha asymmetry (PAA). However, only a limited number of studies investigated PAA, reporting inconsistent results. One study indicated that infants of depressed mothers exhibited greater PAA compared to infants of non-depressed mothers. This same study also revealed that PAA may partially mediate the relationship between maternal depression and infant emotional dysregulation [64]. In general, EEG studies investigating asymmetry often overlook PAA as a marker of emotion regulation although PAA has been shown to be a more stable marker than FAA [77]. Previous research has linked decreased PAA to lower levels of positive affectivity in children [78].

The direction and magnitude of the observed EEG asymmetry varied across studies, potentially due to differences in assessment timing (prenatal vs. postnatal), methodology (e.g., resting state vs. task-related), and the specific measures employed. Future research should standardize assessment protocols to allow for more direct comparison of findings across studies. The discrepancy in the effect of prenatal versus postnatal depression also necessitates further investigation into the precise timing and duration of maternal depressive symptoms in relation to their impact on infant neurodevelopment.

### 4.2. EEG Power and Connectivity

Several studies in our review investigated EEG power [60,61] and connectivity [65], providing additional insights into the neural mechanisms underlying the impact of maternal postnatal depression. Nevertheless, changes in these parameters were not as consistent as the findings related to frontal EEG asymmetry. While some studies found associations between maternal depression and altered EEG power, the overall findings remained limited.

We found that the infants of depressed mothers exhibited modified levels of EEG power in the right-hemisphere occipital region at 1 month of age; yet, these findings were not reported at 3 months, where EEG power differences shifted to the frontal region [60]. The findings were unstable over the 3-month study period, which may be attributed to initial sensory activation in the occipital region, potentially induced by breastfeeding [79]. Derived from power frequency analysis, one study investigated the alpha–delta ratio as a potential marker of neurodevelopment in infants exposed to PPD [61]. Notably, this metric was associated more with prenatal depression than specifically with PPD in the aforementioned study. Similarly, no significant associations were reported between levels of maternal depression and EEG functional connectivity in infants [65].

These inconsistencies may reflect the complex nature of the brain’s response to maternal PPD, potentially affecting multiple neural systems and pathways. The lack of uniformity in findings might also underscore the need for more advanced analytical techniques and a deeper consideration of potential confounding variables (e.g., socioeconomic status and maternal anxiety) that could influence the developmental trajectory of the child. Understanding these nuances will be essential for developing targeted interventions to mitigate the adverse effects of maternal depression on infant neurodevelopment.

### 4.3. Clinical Implications

Our findings suggest a significant need for early intervention and support for mothers experiencing postnatal depression (PPD) to mitigate the potential negative impacts on their infants’ neurodevelopment. Children who experience early-life adversities, such as those associated with maternal postnatal depression—including negative mother–infant interactions, neglect, and inadequate caregiving—may face developmental challenges across multiple domains, such as impaired neural maturation, hormonal dysregulation, and an increased risk of psychopathology [80]. Throughout our review, we have highlighted the risk of increased emotional dysregulation in infants exposed to maternal depression, along with potential biomarkers associated with this risk. These markers may serve as reliable indicators as they were not reported in contexts of positive mother–infant interactions such as involvement during play, engaged interaction styles, or breastfeeding. Thus, early intervention programs, including mental health services, parenting support, and social assistance, can be critical in improving maternal well-being and fostering healthier mother–infant relationships, ultimately supporting positive developmental outcomes [81].

Furthermore, the identification of EEG biomarkers as potential predictors of neurodevelopmental risks underscores the importance of closely monitoring infants born to mothers with PPD. This proactive approach could facilitate early intervention and potentially lead to personalized treatment strategies. Longitudinal studies are necessary to track these neurodevelopmental trajectories into adulthood and to more effectively link early neurophysiological measures with long-term functional outcomes.

### 4.4. Limitations

This systematic review acknowledges the limitations inherent in synthesizing studies with varying methodologies. The heterogeneity of the included studies presents challenges for drawing definitive conclusions. Variations in study design, assessment tools for maternal depression, EEG recording methods, and analytical techniques can introduce biases and limitations. The diversity of assessment tools for maternal depression, combined with reliance solely on self-report data, constrains the strength and generalizability of some findings.

Therefore, future research should prioritize standardized assessment protocols and robust statistical methods to achieve greater internal validity and reduce bias.

Several key areas should be prioritized to enhance our understanding of the impact of maternal postnatal depression on infant neurodevelopment. First, the standardization of methodologies is crucial. This includes consistent assessment tools for maternal depression, EEG data acquisition techniques, and analytical approaches to ensure greater comparability and reliability across studies. Second, longitudinal studies that track neurodevelopmental outcomes from infancy through adolescence are essential to fully elucidate the long-term effects of PPD. Third, a deeper investigation into the underlying neural mechanisms is warranted. This could involve exploring other neurophysiological and neuroimaging techniques to gain a more comprehensive understanding of the brain changes associated with PPD. Finally, the rigorous testing of interventions aimed at mitigating the negative impact of PPD on infant neurodevelopment is vital to inform both clinical practice and public health policy.

## 5. Conclusions

In conclusion, this systematic review has presented evidence of mixed findings regarding a significant association between maternal postnatal depression (PPD) and alterations in infant EEG biomarkers. The inconsistency of findings regarding the association between EEG markers and prenatal or postnatal exposure to maternal depression highlights the complexity of this relationship and underscores the need for further research. Our findings suggest, however, that neural changes may originate with prenatal exposure and are further amplified by postnatal exposure. Consequently, concurrent maternal depression appears to have the most pronounced impact on offspring neural activity. However, the heterogeneity of study designs and methodologies necessitates further research employing standardized protocols to strengthen causal inferences. Future studies should prioritize longitudinal designs to track long-term neurodevelopmental outcomes, investigate the underlying biological mechanisms linking maternal depression to altered EEG patterns, and evaluate the efficacy of targeted interventions aimed at mitigating these negative effects. Only through such comprehensive investigations can we effectively translate these findings into improved clinical practices and enhanced support for mothers and their infants. Ultimately, addressing maternal PPD not only benefits mothers but also fosters healthier developmental trajectories for their children, positioning maternal mental health as a vital component of public health initiatives.

## Figures and Tables

**Table 1:** Characteristics of included studies.

No.	Author, Date	Country	Study Type	Group	Sample Size	Mean Maternal Age (SD)	Married (%)	Primiparity N (%)	Mean Infant Age (SD)	Infant Gender Male N (%)
1	Diego et al., 2004 [11]	USA	Cohort	Total sample	n = 80 mother–infant dyads	27 years (6.5)	63.75%	N/A	1.7 weeks (0.8)	43.00%
2	Diego et al., 2006 [59]	USA	Cohort *	Total sample	n = 66 mother–infant dyads	28 (6.8)	68%	N/A	17 weeks (3.3)	32%
3	Goodman et al., 2021 [58]	USA	Cohort	Total sample	n = 132 women; n = 136 infants	33.75 years (4.35)	91.70%	46%	12 months at enrollment	72 (52.9%)
4	Hardin et al., 2021 [60]	USA	Cohort *	Total sample	n = 113 mother–infant dyads	31 years (4.74)	106 (93.8%) at 1 mo assessment; 78 (96.4%) at 3 mo assessment	43 (38.1%) at 1 mo assessment; 26 (32.1%) at 3 mo assessment	37.14 days (5.88) at 1 mo assessment; 95.36 days (11.39) at 3 mo assessment	45.00%
5	Jones et al., 2004 [55]	USA	Case–control *	Case	n = 31 depressed mother–infant dyads	32.37 years (6.26)	29 (93.5%)	9 (29.03%)	35.99 days (5.7) at 1 mo assessment; 92.66 days (9.19) at 3 mo assessment; reported for total sample	39 (50%) at 1 mo assessment; 34 (54.83%) at 3 mo assessment; reported for total sample
				Control	n = 47 non-depressed mother–infant dyads	32.14 years (5.03)	45 (94.75%)	16 (34.04%)		
6	Kling et al., 2023 [61]	USA	Cohort *	Total sample	n = 92 pregnant women	30.49 (4.22)	N/A	N/A	4.27 months (1.47)	N/A
7	Krzeczkowski et al., 2021 [62]	Canada	Case–control *	Case	n = 40 mother–infant dyads	32.3 years (4.1)	26 (68%)	21 (53%)	5.6 months (2.7)	16 (40%)
				Control	n = 40 mother–infant dyads	32.7 years (5.1)	30 (75%)	22 (55%)	5.9 months (2.6)	16 (40%)
8	Lusby et al., 2014 [57]	USA	Cohort *	Total sample	n = 65 mother–infant dyads	33.8 (4.35)	88%	43.40%	3 months at enrollment	N/A
9	Lusby et al., 2016 [63]	USA	Cohort *	Total sample	n = 234 mother–infant dyads (242 infants)	33.84 (4.49)	88%	43%	3 months at enrollment	126 (52%)
10	Marino et al., 2019 [64]	Italy	Cohort *	Total sample	n = 104 mother–infant dyads	N/A	N/A	N/A	6 months at enrollment	53 (50.96%)
11	Soe et al., 2016 [65]	Singapore	Cohort *	Total sample	n = 258 mother–infant dyads	30.4 (5.1)	N/A	37%	184.5 weeks (6.6) at 6 mo visit; 553.8 weeks (13.7) at 18 mo visit	82 (47.12%) at 6 assessment; 77 (49.04%) at 18 months; 33 (45.2%) for the overlapped sample
12	Wen et al., 2017 [66]	Singapore	Cohort	Total sample	n = 111 mother–infant dyads	30.27 years (4.71)	N/A	N/A	26.39 weeks (0.95)	49 (44.1%)

* Studies marked with * employed a longitudinal design.

**Table 2:** Maternal depression measures and scores.

No.	Author, Date	Depression Measure	Mean Depression Score (SD)	Exposure (Time of Assessment)
1	Diego et al., 2004 [11]	CES-D (Center for Epidemiologic Studies Depression Scale)	a. prepartum–postpartum group: 25.75 (9.19) at prepartum, 23.30 (10.21 at postpartum); b. prepartum only group: 21.9 (6.34) at prepartum, 7.5 (3.36) at postpartum; c. postpartum only group: 9.7 (4.21) at prepartum, 19.05 (10.43) at postpartum; d. non-depressed group 7.25 (3.02) at prepartum, 5.25 (3.48) at postpartum.	Prenatal and postnatal
2	Diego et al., 2006 [59]	CES-D	N/A;	Postnatal
3	Goodman et al., 2021 [58]	Beck Depression Inventory (BDI); Beck Depression Inventory—Second Edition (BDI-II)	8.13 (7.25) at 12 mo infant age	Prenatal and postnatal
4	Hardin et al., 2021 [60]	CES-D	a. 21.5 (6.6) and 9.56 (5.74) for depressed group at 1 mo and 3 mo, respectively; b. 6.57 (1.4) and 4.48 (3.73) for the non-depressed group at 1 mo and 3 mo, respectively.	Postnatal
5	Jones et al., 2004 [55]	CES-D	a. 19 (2.53) and 7.5 (4.32) for breastfed infants at 1 mo and 3 mo, respectively; b. 19.27 (6.11) and 16.9 (6.48) for bottle-fed infants at 1 mo and 3 mo.	Postnatal
			a. 5.07 (3.28) and 4.54 (3.44) for breastfed infants at 1 mo and 3 mo, respectively; b. 6.5 (3.45) and 3.73 (3.39) for bottlefed infants at 1 mo andd 3 mo, respectively.	
6	Kling et al., 2023 [61]	EPDS (Edinburgh Postnatal Depression Scale)	a. 2nd trimester depressive symptoms (n = 77): M = 6.39 (4.12); b. 3rd trimester depressive symptoms (n = 82): M = 5.46 (4.74); c. postnatal depression symptoms (n = 67): M = 5.30 (4.26).	Prenatal and postnatal
7	Krzeczkowski et al., 2021 [62]	EPDS	14.7 (5.4)	Prenatal and postnatal
			4.6 (3.4)	
8	Lusby et al., 2014 [57]	BDI, BDI-II	N/A;	Prenatal and postnatal
9	Lusby et al., 2016 [63]	BDI	a. prenatal maternal depressive symptoms: M = 9.01 (6.41); b. postpartum maternal depressive symptoms: 8.68 (6.77).	Prenatal and postnatal
10	Marino et al., 2019 [64]	Adult Self-Report, Achenbach System of Empirically Based Assessment	7.26 (4.89)	Postnatal
11	Soe et al., 2016 [65]	EPDS	a. prenatal maternal depressive symptoms: 7.6 (4.4) at 6 mo assessment, 7.9 (4.4) at 18 mo assessment; 7.6 (4.2) for the overlapped sample; b. postnatal maternal depressive symptoms: 6.8 (4.8) at 6 mo; 6.4 (4.5) at 18 mo; 6.6 (4.6) for the overlapped sample.	Prenatal and postnatal
12	Wen et al., 2017 [66]	EPDS	7.29 (4.42) prenatal score; 6.59 (4.75) postnatal score	Prenatal and postnatal

**Table 3:** Infant EEG metrics and findings.

Nr	Author, Date	Acquisition	Reference	Epoch Duration	EEG Metric	Results
1	Diego et al., 2004 [11]	3 min, baseline recording; 4 channels	Cz	N/A	EEG asymmetry	The 1-week-old offspring of prepartum–postpartum depressed mothers, t (33) = 4.14, *p* < 0.01, and prepartum depressed mothers, t (33) = 2.91, *p* < 0.05, exhibited greater relative right frontal EEG asymmetry than the infants of non-depressed mothers. The 1-week-old offspring of prepartum–postpartum depressed mothers, t (32) = 3.06, *p* < 0.05, exhibited greater relative right frontal EEG asymmetry than the infants of postpartum depressed mothers.
2	Diego et al., 2006 [59]	3 min resting state, 4 channels	Cz	N/A	EEG asymmetry (FAA, PAA)	Infants exposed to postnatal depression exhibited greater relative right frontal EEG asymmetry than infants of non-depressed mothers (*p* < 0.06 for all comparisons) at 3–6 mo. Mother–infant interaction played a role, with infants of withdrawn depressed mothers exhibiting greater relative right frontal EEG asymmetry than infants of intrusive depressed mothers (*p* < 0.06) at 3–6 mo. No significant differences in parietal EEG asymmetry were noted.
3	Goodman et al., 2021 [58]	3 min baseline/bubbles, 2 min peek a-boo, 5 min play, 5 min feeding, and 5 min distract task; 16 channels	Cz	N/A	EEG asymmetry	Infants of mothers who had higher prenatal depression exhibited greater right frontal EEG asymmetry (b = −0.002, *p* = 0.02) at 12 mo. There was a significant positive effect of postnatal depression on the asymmetry score (b = 0.002, *p* = 0.05), indicating that infants exposed to higher postnatal depression exhibited more left frontal EEG asymmetry compared to infants of mothers suffering from prenatal or concurrent prenatal-postnatal depressed mothers.
4	Hardin et al., 2021 [60]	5 min, baseline recording; 8 channels	Cz	N/A	EEG asymmetry EEG power	(1) EEG asymmetry: Main effect of depression group on regional EEG asymmetry was noted at 1 month (F(1, 86) = 4.16, *p* = 0.04, η² = 0.05), with depressed infants showing overall greater relative right asymmetry (M = −0.56, SD = 0.32). Breastfed infants of depressed mothers exhibited left frontal asymmetry while bottle-fed infants exhibited right frontal asymmetry at 3 mo. (2) EEG power: Bottle-fed infants of depressed mothers showed higher right hemisphere and left hemisphere occipital EEG power at 1 mo (M = 4.31, SD = 1.51 for right hemisphere; M = 4.15, SD = 1.19 for left hemisphere) compared to infants of bottle-fed depressed mothers (M = 2.08, SD = 1.43 for right hemisphere; M = 2.61, SD = 1.54 for left hemisphere). Bottle-fed infants of depressed mothers show lower left hemisphere frontal EEG power, as well as greater right hemisphere frontal EEG power, compared to breastfed infants of depressed mothers at 3 mo. No other EEG-power between-group differences were noted.
5	Jones et al., 2004 [55]	5–6 min, resting state, eyes-open; 8 channels	Cz	N/A	EEG asymmetry	Greater relative frontal EEG asymmetry was significantly correlated with depression score (r = −0.299, *p* = 0.008). Maternal depression was strongly predictive of infant frontal EEG asymmetry (direct effect = −0.30, t = 2.73, *p* < 0.05) at 1–3 mo. Breastfed infants exposed to maternal postnatal depression and infants from non-depressed mothers did not show differences in frontal EEG asymmetry (*p* > 0.05).
6	Kling et al., 2023 [61]	5 min resting state, 21 channels; 2048 Hz	average reference	N/A	alpha–delta ratio	Higher depressive symptoms in the second trimester were associated with a smaller alpha–delta ratio at parietal electrodes (B = 0.07, SE(B) = 0.02, 95% CI [0.11, −0.03], *p* < 0.01) at 4 mo of age; higher depressive symptoms in the third trimester (B = 0.05, SE(B) = 0.02, 95% CI [0.01, 0.10], *p* = 0.02) were associated with larger alpha–delta ratio scores at parietal electrodes at infant 4 mo of age. Postnatal depressive symptoms (B =0.02, SE(B) = 0.03, 95% CI [0.03, 0.07], *p* = 0.49) were unrelated to parietal alpha–delta ratio scores. No association between maternal depression (prenatal/postnatal) and alpha–delta ratio at frontal electrodes in infants was noted.
7	Krzeczkowski et al., 2021 [62]	5 min, resting state, eyes-open, 128 channels, 250 Hz	Cz	2 s	EEG asymmetry (FAA)	Infants of depressed mothers differed significantly in FAA (M = −0.091, SD = 0.31) compared to infants of non-depressed mothers (M = 0.13, SD = 0.35, *p* = 0.005, d = 0.67) at age of enrollment (<12 months).
8	Lusby et al., 2014 [57]	3 min baseline, 5 min feeding, and 5 min play segment; 16 channels	Cz	N/A	EEG asymmetry	No significant association between infant baseline frontal EEG asymmetry scores and (1) maternal concurrent depression r(65)= −0.06, *p* = 0.63, (2) maternal postpartum depression r(65) = 0.05, *p* = 0.71 at 3 mo of age was noted. No significant association between infant baseline frontal EEG asymmetry scores and (1) maternal concurrent depression r(64)= −0.16, *p* = 0.21, (2) maternal postpartum depression r(66) = 0.01, *p* = 0.95 at 6 mo of age was noted. Prenatal depressive symptoms and infant EEG asymmetry scores were significantly associated among women with high postpartum depressive symptoms, r(26) = −0.44, *p* = 0.01
9	Lusby et al., 2016 [63]	3 min resting state, 16 channels	Cz	N/A	EEG asymmetry (FAA)	Infants of mothers with high prenatal depressive symptoms showed a significant shift in EEG asymmetry over time (β = 0.06, SE = 0.02, *p* = 0.01): higher negative affect (NA) was associated with greater right frontal EEG asymmetry, with a reversed pattern by 12 months of age. Infants of mothers with depression in either the prenatal or postpartum period showed a shift in EEG asymmetry patterns over time (β = −0.07, SE = 0.04, *p* = 0.06, marginal significance): lower relative right EEG asymmetry at 3 mo, followed by greater relative right frontal EEG asymmetry at 12 mo. Infants of mothers with depression in both the prenatal and postpartum periods showed the opposite trajectory (β = 0.05, SE = 0.02, *p* = 0.03). EEG asymmetry trajectories differed depending on whether maternal depression was present in one period (prenatal or postpartum) or in both periods (β = 0.12, SE = 0.04, *p* = 0.004).
10	Marino et al., 2019 [64]	3 min resting state; 60 channels; 250 Hz	Cz	1 s	EEG asymmetry (FAA, PAA)	Maternal depression symptoms were significantly positively correlated with higher FAA (r = 0.21, *p* = 0.04) and PAA (r = 0.23, *p* = 0.02) at 6 mo. Maternal depression symptoms had a significant indirect effect on child dysregulation via PAA (β = 0.065, SE = 0.033, 95% CI [0.001, 0.139], *p* = 0.048), but not via FAA (β = −0.008, SE = 0.018, 95% CI [−0.057, 0.042], *p* = 0.672).
11	Soe et al., 2016 [65]	40 min passive auditory oddball task (at 6 and 18 mo); 99 channels; 250 Hz	Cz	N/A	EEG asymmetry Functional connectivity	No association of postnatal EPDS with either bilateral frontal activity (β = −0.045, *p* > 0.05 at 6 mo, β = −0.033 *p* > 0.05 at 18 mo for left hemisphere; β = −0.073, *p* > 0.05 at 6 mo, β = −0.046, *p* > 0.05 at 18 mo for right hemisphere) and asymmetry (β = −0.051, *p* > 0.05 at 6 mo, β = 0.023, *p* > 0.05 at 18 mo) and frontal connectivity (β = −0.004, *p* > 0.05 at 6 mo, β = 0.047, *p* > 0.05 at 18 mo for left hemisphere; β = −0.016, *p* > 0.05 at 6 mo, β = −0.048, *p* > 0.05 at 18 mo) was noted at 18 mo in 6-month and 18-month-old infants. No association of prenatal EPDS with either bilateral frontal activity and asymmetry or functional connectivity was noted. An increase in depressive symptoms from the prenatal to postnatal time predicted greater right frontal activity and relative right frontal asymmetry at 6 months of age (β = −0.205, *p* < 0.01) but not at 18 months of age (β = −0.047, *p* > 0.05).
12	Wen et al., 2017 [66]	2 min baseline, 38 min passive auditory oddball task; 99 channels; 250 Hz	Cz	2 s	EEG asymmetry (FAA)	Greater relative right frontal EEG asymmetry was associated with higher levels of postnatal depression (β = −0.283, df = 48, *p* = 0.04) and lower maternal sensitivity (β = 0.243, df = 48, *p* = 0.04) in infants aged 6 mo.

## Supplementary Materials

The following supporting information can be downloaded at: https://www.mdpi.com/article/10.3390/children12040396/s1, Supplementary File S1. Search Strings; Supplementary File S2. Newcastle–Ottawa Scale (NOS).
