# Supplementary material for: Neurodevelopmental Impact of Maternal Postnatal Depression: A Systematic Review of EEG Biomarkers in Infants
Source: Children (Basel). 2025 Mar 21;12(4):396. doi: 10.3390/children12040396 (PMC12026314; doi:10.3390/children12040396)
Supplement: Supplementary file 1 [file children-12-00396-s001.zip › Supplementary File S1. Search Strings.pdf]

Supplementary File S1. Search Strings.

1. **Web Of Science**

(TS=(newborn) OR TS=(neonat*) OR TS=(baby) OR TS=(babies) OR TS=(infant*) OR TS=(child*)) AND (TS=(electroencephalograph*) OR TS=("electro-encephalograph*") OR TS=(EEG) OR TS=(somnograph*)) AND (TS=(postpartum depression) OR TS=(postnatal depression))*

2. **PubMed/MEDLINE**

("newborn"[Title/Abstract] OR "neonat*"[Title/Abstract] OR "baby"[Title/Abstract] OR "babies"[Title/Abstract] OR "infant*"[Title/Abstract] OR "child*"[Title/Abstract]) AND ("electroencephalogra*"[Title/Abstract] OR "electro encephalograph*"[Title/Abstract] OR "EEG"[Title/Abstract] OR "somnograph*"[Title/Abstract]) AND ("postpartum depression"[Title/Abstract] OR "postnatal depression"[Title/Abstract])*

3. **SCOPUS**

(TITLE-ABS-KEY (newborn OR neonat* OR baby OR babies OR infan* OR child*) AND TITLE-ABS-KEY (eeg OR electroencephalograph* OR "electro-encephalograph*" OR somnograph*) AND TITLE-ABS-KEY (postpartum AND depression OR postnatal AND depression))*
