# Supplementary material for: Neurodevelopmental Impact of Maternal Postnatal Depression: A Systematic Review of EEG Biomarkers in Infants
Source: Children (Basel). 2025 Mar 21;12(4):396. doi: 10.3390/children12040396 (PMC12026314; doi:10.3390/children12040396)
Supplement: Supplementary file 1 [file children-12-00396-s001.zip › Supplementary File S2 -Newcastle Ottawa Scale NOS.pdf]

Newcastle Ottawa Scale - for Cohort Studies

Selection:

1) Representativeness of the exposed cohort:
   a) Truly representative of the average in target population (all subjects or random sampling). *
   b) Somewhat representative of the average in the target population (non-random sampling). *
   c) Selected group of users.
   d) No description.

2) Selection of the non-exposed cohort:
   a) Drawn from the same community as the exposed cohort. *
   b) Drawn from a different source.
   c) No description.

3) Ascertainment of exposure:
   a) Secure record (e.g., surgical records). *
   b) Structured interview. *
   c) Written self-report.
   d) No description.

4) Demonstration that outcome of interest was not present at start of study:
   a) Yes. *
   b) No.

Comparability:

1) Comparability of cohorts on the basis of the design or analysis:
   a) Study controls for gender/sex and age. *
   b) Study controls for any additional factor (e.g., depression, anxiety etc). **
   c) Not comparable on the basis of the design or analysis.

Outcome:

1) Assessment of outcome:
   a) Independent blind assessment. *
   b) Record linkage. *
   c) Self-report/interview.
   d) No description.

2) Was follow-up long enough for outcomes to occur:
   a) Yes (e.g., 6 months or over). *
   b) No.

3) Adequacy of follow up of cohorts:
   a) Complete follow up - all subjects accounted for. *

b) Subjects lost to follow up but rate given (description given and <30%) *
c) Subjects lost to follow up (no description given and <70%).
d) No description at all.

Newcastle-Ottawa Scale - for Case-Control Studies

Selection

1) Is the case definition adequate?
   a) yes, with independent validation *
   b) yes, e.g. record linkage or based on self reports
   c) no description

2) Representativeness of the cases
   a) consecutive or obviously representative series of cases *
   b) potential for selection biases or not stated

3) Selection of Controls
   a) community controls *
   b) hospital controls
   c) no description

4) Definition of Controls
   a) no history of disease (endpoint) *
   b) no description of source

Comparability
1) Comparability of cases and controls on the basis of the design or analysis
   a) study controls for fracture risk factors (age) *
   b) study controls for any additional factor * (other fracture risk factors)

Exposure
1) Ascertainment of exposure
   a) secure record (verified fracture) *
   b) structured interview where blind to case/control status *
   c) interview not blinded to case/control status
   d) written self report or medical record only
   e) no description

2) Same method of ascertainment for cases and controls
   a) yes *
   b) no

3) Non-Response rate
   a) same rate for both groups *

b) non respondents described
c) rate different and no designation

Supplementary Table S1. NOS for cohort studies.

Author (year)	Selection				Comparability	Outcome			Total Score
	Q1	Q2	Q3	Q4		Q1	Q2	Q3	
Diego et al. (2004)	-	★	-	★	★★	★	-	★	6
Diego et al. (2006)	-	★	-	★	★★	★	★	★	7
Goodman et al. (2021)	-	★	-	★	★★	★	★	★	7
Hardin et al. (2021)	-	★	-	★	★	★	-	★	5
Kling et al. (2023)	★	★	-	★	★★	★	★	-	7
Lusby et al. (2014)	-	★	-	★	★	★	★	★	6
Lusby et al. (2016)	-	★	-	★	★★	★	★	★	7
Marino et al. (2019)	★	★	-	★	★★	★	★	★	8
Soe et al. (2016)	★	★	-	★	★★	★	★	-	7
Wen et al. (2017)	★	★	-	★	★★	★	★	-	7

Supplementary Table S2. NOS for Case-Control Studies

Author (year)	Selection				Comparability	Outcome			Total Score
	Q1	Q2	Q3	Q4		Q1	Q2	Q3	
Jones et al. (2004)	-	-	★	-	★	-	★	-	3
Krzeczkowski et al. (2021)	★	★	★	★	★★	-	★	-	7
